# Parsimonious Logistic Regression Models for 90-Day Mortality Prediction in the Intensive Care Unit

**DOI:** 10.7759/cureus.97940

**Published:** 2025-11-27

**Authors:** Sadiq A Olayinka

**Affiliations:** 1 General Internal Medicine, Walsall Manor Hospital, Walsall, GBR

**Keywords:** apache ii, intensive care unit, logistic regression, mortality prediction, sofa score

## Abstract

Introduction

Early identification of critically ill patients is essential in intensive care units to guide triage and allocate resources. To support these decisions, prognostic scoring systems are commonly used to estimate illness severity and predict outcomes. The Acute Physiology and Chronic Health Evaluation II (APACHE II) and the Sequential Organ Failure Assessment (SOFA) are among the most widely used. While newer APACHE versions (III and IV) offer enhanced predictive power, they require extensive variables, are computationally expensive, and often rely on proprietary systems - making them less feasible for routine emergency care. In contrast, APACHE II and SOFA are simple, transparent and can be calculated using spreadsheets, clinical apps, or bedside tools. These advantages make them ideal for real-time use in busy and resource-limited settings. While each system provides valuable prognostic information individually, combining them may yield a more comprehensive and accurate prediction of mortality risk. The primary outcome in this study was 90-day mortality following ICU admission, to capture both early and delayed deaths. The objective was to develop univariate and multivariate logistic regression (LGR) models using SOFA and APACHE II scores. The study also aimed to identify optimal probability threshold values with the Youden J statistic to improve mortality risk classification in the first 24 hours of ICU admission.

Method

This was a retrospective single-centre study including ICU admissions at Walsall Manor Hospital between January 2024 and May 2025. SOFA and APACHE II scores calculated within 24 hours of admission were stratified into a development set (January-December 2024) and an independent test set (January-May 2025). Univariable and multivariable logistic regression models were developed using these scores as predictors. Two resampling approaches - bootstrapping and random 80/20 data splits - were applied to identify optimal probability thresholds using Youden’s J statistic. Model performance was evaluated in the independent test cohort in terms of discrimination, calibration, and classification metrics at both the conventional (0.50) and optimized thresholds.

Results

In the independent test cohort (n = 281), all models showed low sensitivity at the conventional 0.50 probability cut-off (sensitivities: SOFA 0.29, APACHE II 0.27, Combined 0.35), misclassifying more than half of deaths as survivors. Optimized probability thresholds were substantially lower (probabilities: SOFA ~0.20, APACHE II ~0.27, Combined ~0.19) and improved sensitivity to 0.69, 0.78, and 0.88, respectively. Specificity and positive predictive values were modest (0.60 - 0.74 and 0.30 - 0.40, respectively), reflecting increased false positives. However, negative predictive values remained high (0.91 - 0.96), meaning patients classified as low risk almost always survived.

Conclusion

SOFA and APACHE II are well-established ICU severity scores, but reliance on the conventional 0.50 probability cut-off in logistic regression markedly reduces sensitivity and risks under-detecting patients at risk of mortality. In this study, optimising probability thresholds substantially improved sensitivity while maintaining consistently high negative predictive values. Positive predictive values remained modest, indicating that high-risk predictions should be interpreted as signals for closer clinical attention rather than definitive indicators of impending mortality.

## Introduction

Outcome prediction in intensive care units (ICU) is crucial for patient triage, communication and intervention planning. As far back as 1985, Knaus et al introduced a robust scoring system - Acute Physiology and Chronic Health Evaluation II (APACHE II) to assess the severity of disease in adult ICU patients and predict hospital mortality [1]. APACHE II consisted of 12 commonly measured variables, which are indicators of physiology, age, and chronic health.

Since its development, however, ICU practices have changed significantly. This has necessitated refinements of APACHE to APACHE III and, more recently, APACHE IV [2-3]. Each later iteration involves increasing the number of variables. APACHE III has 20 physiological variables, while APACHE IV has 142 variables. While the prognostic values of these later tools are much better, they are not readily accessible and require both significant computational power and proprietary scoring algorithms. APACHE II, on the other hand, can be calculated manually and coded with simple, readily available tools such as spreadsheet macros.

Another tool widely used in ICUs is the Sequential Organ Failure Assessment (SOFA) [4]. This tool was introduced in 1996 as a standardized tool to describe and quantify organ dysfunction in ICU patients. It evaluates six organ systems using parameters representative of respiratory, coagulation, liver, cardiovascular, central nervous system and renal system. Unlike APACHE II, which is measured in the first 24 hours, SOFA scores are repeated every 24 hours. It is a simple, repeatable way to assess organ dysfunction over time. Similar to APACHE II, it is also a simple tool which can be coded easily in spreadsheet tools. For direct comparison purposes, the SOFA being referenced in this study was that recorded 24 hours after ICU admission.

Individually, each of these tools has limitations in sensitivity and generalizability. SOFA emphasizes acute organ dysfunction, and it is sensitive to short-term physiological changes; however, it omits age and chronic morbidity, which may lead to underestimation of baseline risk. APACHE II, on the other hand, incorporates age and chronic health status, but may be less responsive to chronic clinical deterioration. Combining them may enhance prognostic accuracy by leveraging complementary information.

The objective of this study was to evaluate the predictive performance of SOFA, APACHE II, and a combined SOFA-APACHE II model in estimating 90-day all-cause mortality following ICU admission using logistic regression. A 90-day horizon was chosen to capture both early and delayed deaths, providing a more comprehensive assessment of prognosis than 30-day mortality, which may underestimate the true burden of critical illness [5,6]. Logistic regression was selected over time-to-event approaches such as Cox proportional hazards because the outcome was defined as a fixed 90-day vital status (alive or deceased), and complete follow-up was available for all patients through linkage with the national shared care record.

## Materials and methods

Study design and data collection

**Figure 1:**
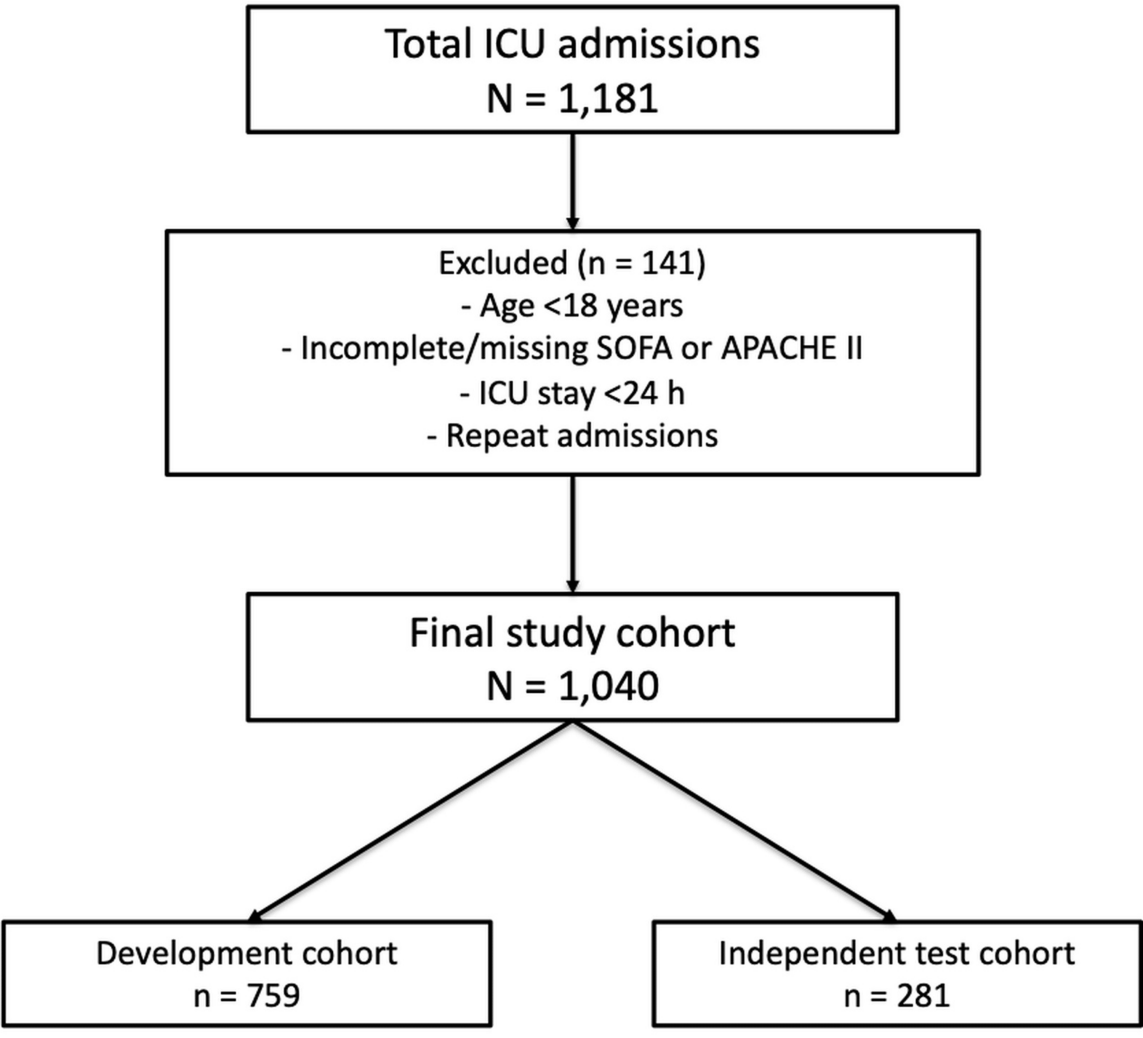
Patient selection flow diagram. A total of 1,181 ICU admissions were screened between January 1, 2024, and May 31, 2025. After applying inclusion and exclusion criteria, 1,040 first admissions were retained for analysis. Of these, 759 were assigned to the development cohort (January–December 2024) and 281 to the test cohort (January–May 2025).

This retrospective study, part of an audit, included patients admitted to Walsall Manor Hospital ICU from January 1st, 2024, to May 31st 2025. Patients were divided into two cohorts: development cohort (admitted between January 1st 2024, and December 31st 2024) and test cohort (admitted between January 1st 2025, and May 31st 2025). This temporal split was intentionally designed to simulate real-world prospective use. Specifically, all models were trained exclusively on admissions from January to December 2024, and these fitted models were then applied to patients admitted in 2025. This mirrors the clinical scenario in which a prediction model is developed on historical data and subsequently used to estimate risk in future patients without being retrained. During the study period, there were no major changes in ICU admission criteria, staffing structure, or core treatment protocols that would be expected to substantially alter case-mix or outcomes. The patient selection process is illustrated in Figure 1, while inclusion and exclusion criteria are summarized in Table 1.

**Table 1:** Inclusion and Exclusion Criteria

Inclusion Criteria	Rationale	Exclusion Criteria	Rationale
Adults (≥18 years)	Ensures clinical comparability and applicability of severity scores	Patients <18 years	Paediatric ICU patients use different scoring systems
ICU admission between Jan 1, 2024, and May 31, 2025	Defines study period	Incomplete or missing SOFA/APACHE II data	Ensures consistent model input
SOFA and APACHE II scores recorded within 24 h of admission	Standardizes timing of severity scoring	ICU stay <24 h (discharge/transfer)	Insufficient time to collect reliable data
Known outcome at 90 days post-admission	Enables valid outcome modelling	Repeat ICU admissions from the same patient	Only the first admission was included to ensure independence of observations

**Figure 2:**
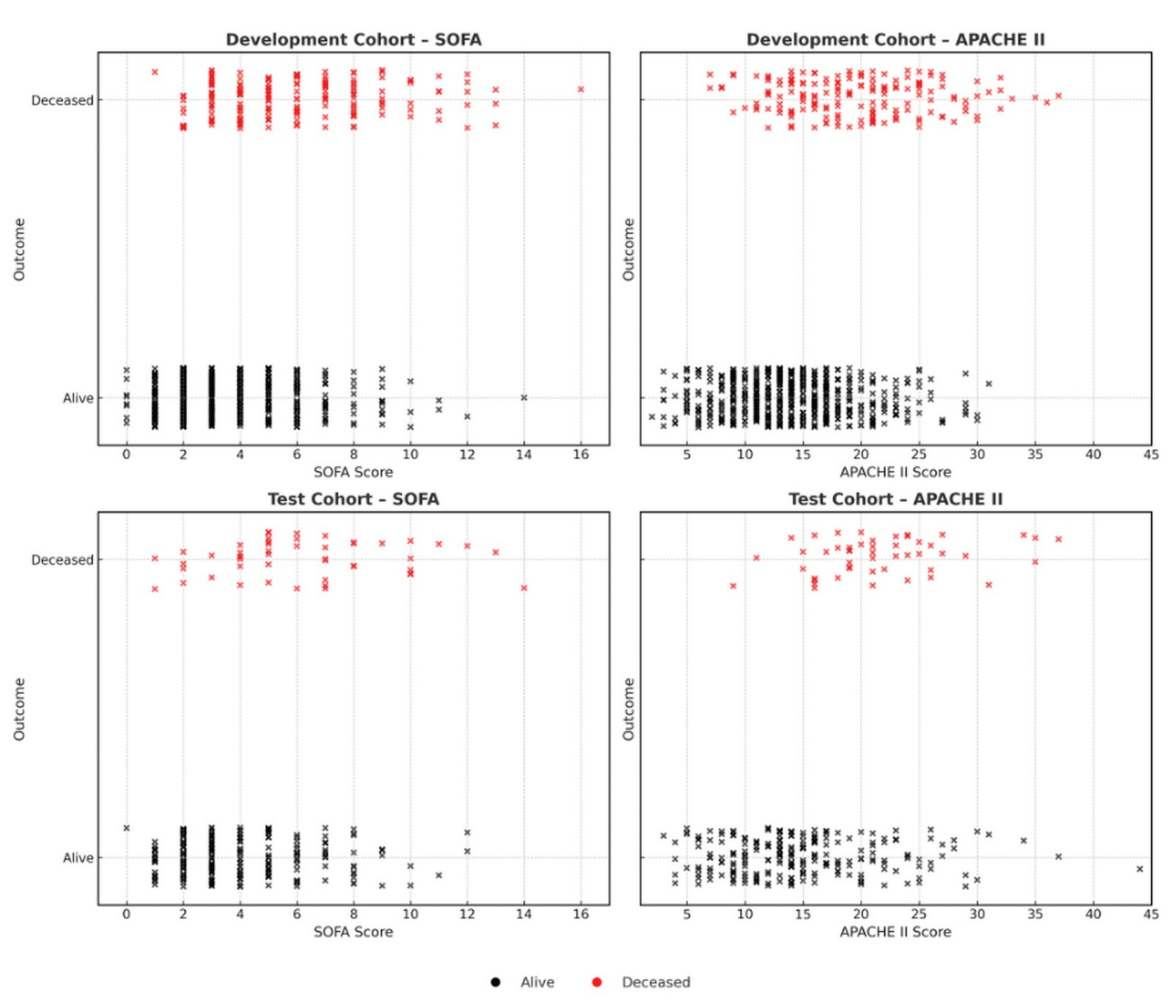
Distribution of SOFA and APACHE II scores in development and test cohorts. Scatterplots showing SOFA scores (left panels) and APACHE II scores (right panels) against 90-day outcomes in the development cohort (top row) and independent test cohort (bottom row). Each point represents a patient; black marks indicate survivors and red marks indicate non-survivors. SOFA: Sequential Organ Failure Assessment; APACHE II: Acute Physiology and Chronic Health Evaluation II

Physiological variables required to generate SOFA and APACHE II scores were obtained from bedside ICU observation charts and the Walsall Manor Hospital Electronic Patient Record System (FUSION). These data were subsequently imported into Medicus Critical Care (Mela Solutions Ltd), a licensed electronic medical record platform, which automatically generated SOFA and APACHE II scores for each patient. Access to the system is administered by the hospital, and licensing for these scoring methods is understood to be held by the vendor. The investigator did not manually calculate or reproduce either score. Figure 2 displays jittered scatter plots of SOFA and APACHE II scores. 

Outcome measure

The primary outcome was 90-day in-hospital mortality following ICU admission. Survival status was determined from hospital records. A 90-day timeframe was selected because it captures delayed deaths that may be missed at 30 days and provides a more accurate reflection of illness severity, ICU care, and early post-discharge trajectory [5,6].

Predictors

Two predictor variables were evaluated: the SOFA score and the APACHE II score. All physiological and laboratory parameters recorded within the first 24 hours of ICU admission were used for score generation. Bedside observations were documented contemporaneously by nurses and doctors as part of routine clinical care, while laboratory investigations (e.g., bilirubin, creatinine, platelets, arterial blood gases) were automatically stored in the hospital’s laboratory information system. Additional information relevant to scoring, such as vasopressor or inotrope use, ventilatory support, and other organ-support interventions during the same 24-hour period, was recorded in the ICU clinical charts.

The ICU clinical data team reviewed all available clinical charts and electronic records and identified the most deranged (worst) recorded value for each scoring component, consistent with the original SOFA and APACHE II definitions. These verified worst values were entered into the hospital’s critical care information system (Medicus Critical Care, Mela Solutions Ltd), which calculated the total SOFA and APACHE II scores using the original published scoring rules [1,4]. When a required variable was missing, the data team could mark the field as “missing.” Medicus does not impute or estimate missing values; instead, missing items default to a normal score, reflecting standard scoring convention and preventing artificial inflation of severity.

No retrospective recalculation or modification of these clinician-verified scores was performed for this study. The SOFA score is freely available for academic use (Appendix 1), while APACHE II scoring was generated through the licensed Medicus module (Appendix 2). Univariable logistic regression models were fitted for each score separately, and a multivariable model combining SOFA and APACHE II was evaluated to determine whether integrating the two systems improved predictive performance.

Statistical analysis

Statistical analyses were performed with open-source Python code on anonymized, de-identified patient data and Microsoft Excel. Statistical significance was assessed using two-sided p-values, with p < 0.05 considered significant. Logistic regression, the standard approach for modelling binary categorical outcomes, was applied [7]. Logistic regression was chosen because the study outcome was a fixed 90-day mortality status rather than a variable survival time. Three models were generated: a SOFA-only model, an APACHE II-only model and a combined model incorporating both scores. The logistic regression model was defined as: p = 1/(1 + exp(-(β₀ + β₁.SOFA Score + β₂. APACHE II Score))). where p represents the predicted probability of 90-day mortality, β₀ is the intercept, and β₁ and β₂ are regression coefficients. For the univariable models, the equation included only one predictor term.

Two resampling approaches were used to estimate probability thresholds: repeated 80/20 random splits and non-parametric bootstrapping [8]. In the first approach, 10,000 random runs were performed; in each run, 80% of the development cohort was randomly selected for training, and the remaining 20% was used for validation. In the second approach, 10,000 non-parametric bootstrap samples were generated by sampling with replacement from the full development cohort, preserving the original sample size in each resample. In each bootstrap iteration, the logistic regression model was refitted on the resampled (in-bag) data and evaluated on the corresponding out-of-bag observations using the same model specification as the primary analysis.

Traditionally, univariate logistic regression models incorporating these prognostic scores have been used to assign probabilities which are translatable to the risk of death. The conventional practice is to use a default threshold probability of 0.50 [7]. However, in imbalanced populations, such as those obtained in the ICU, this can lead to loss of sensitivity and result in underprediction of mortality. To address this, Youden’s J statistic was used to derive optimal thresholds, defined as: J = sensitivity + specificity - 1, with values ranging from 0 (worthless test) to 1 (perfect test) [9].

The Python script implementing these Youden J-based threshold optimization procedures (including both repeated 80/20 splits and bootstrap resampling) is openly available at https://github.com/EndrSadiq/ICU_Logistic_Regression_Model/tree/main.

Model Discrimination was assessed using the area under the receiver operating characteristic curve (AUC)[10]. To quantify uncertainty, 95% confidence intervals (CIs) for the AUC were obtained using a percentile bootstrap. The 2.5th and 97.5th percentiles of the bootstrap distribution were taken as the CI bounds.

Calibration was assessed using the Brier score and the Hosmer-Lemeshow (H-L) goodness-of-fit test [11,12]. The Brier score measures the accuracy of probabilistic predictions for binary outcomes, with values ranging from 0 (perfect accuracy) to 1 (complete inaccuracy). The H-L test evaluates calibration by comparing predicted and observed event rates within subgroups of patients using a chi-square statistic. A non-significant p-value indicates good calibration, whereas a significant p-value suggests a lack of fit.

To evaluate whether the temporal split introduced bias and to simulate prospective use of the models, temporal robustness was assessed using a rolling-origin validation design. Sequential 6-month training windows followed by 2-month testing windows were advanced monthly across the study period (January 2024-May 2025). In each window, the model was trained only on data available up to time k and evaluated on patients admitted thereafter, reflecting real-world deployment. Discrimination (AUC) and calibration (Brier score) were calculated for each test window to quantify performance stability over time.

Ethical approval

This study was conducted as part of an internal clinical audit. The hospital’s Ethics Review Division confirmed that Health Research Authority (HRA) and Research Ethics Committee (REC) approvals were not required because the project involved analysis of routinely collected data and did not introduce or evaluate any change in patient care. All data were anonymized prior to analysis.

## Results

Study population

A total of 1,181 admissions were recorded for the study period. After applying inclusion and exclusion criteria (Table 1), 1040 first admissions were retained for analysis. Seven hundred fifty-nine unique admissions were included in the development cohort and 281 in the test cohort (Figure 1).

In the development cohort, the mean age was 60.7 ± 17.7 years (range 18-93), and 375 patients (49.4%) were female. At 90 days post-ICU admission, 21.8% of the patients had died, while 78.2% were still alive. Here, “alive” refers to patients who were confirmed to be living 90 days after their index ICU admission, irrespective of whether they had been discharged or remained hospitalized. Mortality status was determined from the hospital’s electronic medical record system linked to national Summary Care Records, enabling identification of both in-hospital and post-discharge deaths to ensure complete 90-day follow-up.

In the test cohort, the mean age was 59.9 ± 17.5 years (range 18-92), with 130 patients (46.3%) female. At the follow-up cut-off, 17.4% had passed away, with 82.6% surviving.

The baseline demographic and clinical characteristics are summarized in Table 2. The distribution of SOFA and APACHE II scores between survivors and non-survivors is illustrated in Figure 2.

**Table 2:** Baseline demographic and clinical characteristics of the development and test cohorts Values are presented as mean ± standard deviation (SD) or number (percentage). SOFA: Sequential Organ Failure Assessment; APACHE II: Acute Physiology and Chronic Health Evaluation II

Variable	Development Cohort (n = 759)	Test Cohort (n = 281)
Age, mean ± SD (years)	60.7 ± 17.7	59.9 ± 17.5
Sex, n (%)		
- Male	384 (50.6)	151 (53.7)
- Female	375 (49.4)	130 (46.3)
SOFA score, mean ± SD	4.0 ± 2.5	4.3 ± 2.6
APACHE II score, mean ± SD	15.1 ± 6.0	15.9 ± 7.0
90-day mortality, n (%)	167 (22.0)	49 (17.4)

**Figure 3:**
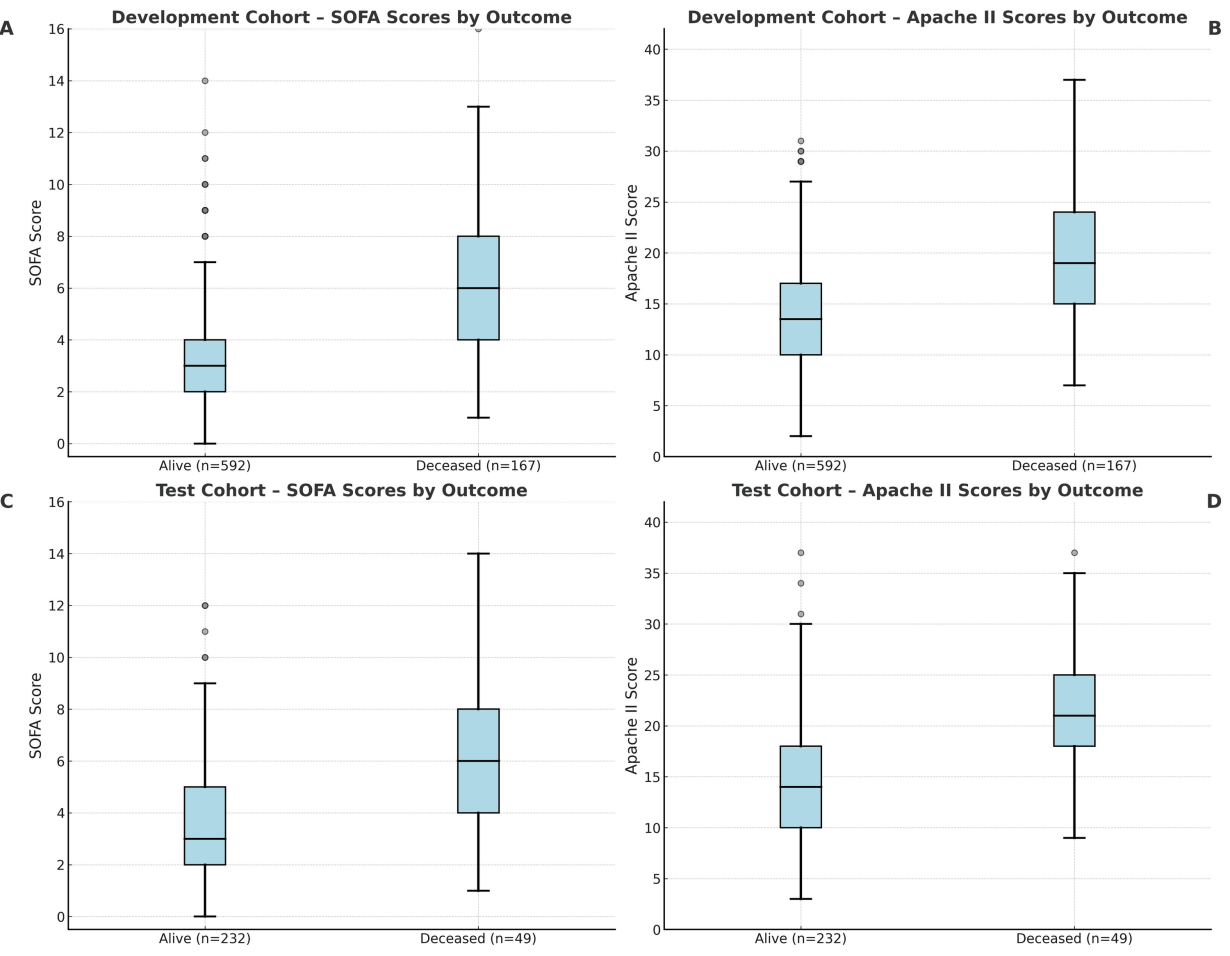
Distribution of SOFA and APACHE II scores by survival status in development and test cohorts. Boxplots show the distribution of Sequential Organ Failure Assessment (SOFA) and Acute Physiology and Chronic Health Evaluation II (APACHE II) scores between survivors (Alive) and non-survivors (Deceased) at 90 days following ICU admission. In the development cohort, SOFA scores are shown in Panel A and APACHE II scores in Panel B. In the test cohort, SOFA scores are shown in Panel C and APACHE II scores in Panel D. Boxes represent the interquartile range with the median indicated by a horizontal line; whiskers denote ranges excluding outliers. Sample sizes for each group are shown on the x-axis.

Model performance

Logistic Regression Models

Regression coefficients for the SOFA, APACHE II and combined models are presented in Table 3. In the univariate models, both SOFA and APACHE II scores showed significant positive associations with 90-day mortality. In the combined model, both SOFA and APACHE II scores remained significant contributors.

**Table 3:** Logistic regression coefficients, odds ratios, and p-values for 90-day mortality prediction Logistic regression coefficients (β), standard errors (SE), odds ratios (OR) with 95% confidence intervals (CI), and p-values are shown for models based on SOFA, APACHE II, and their combination. Odds ratios indicate the multiplicative change in odds of 90-day mortality for each one-unit increase in the predictor.

Model	Predictor	Beta Coefficient	Std Error	Odds Ratio	p-value
SOFA only	Intercept	-3.12	0.22	—	—
	SOFA	0.41	0.04	1.51 (1.39–1.63)	< 0.001
APACHE II only	Intercept	-4.14	0.32	—	—
	APACHE II	0.17	0.02	1.19 (1.15–1.23)	< 0.001
Combined (SOFA+APACHE II)	Intercept	-4.37	0.34	—	—
	SOFA	0.28	0.05	1.33 (1.21–1.45)	< 0.001
	APACHE II	0.11	0.02	1.12 (1.07–1.16)	< 0.001

Discrimination

**Figure 4:**
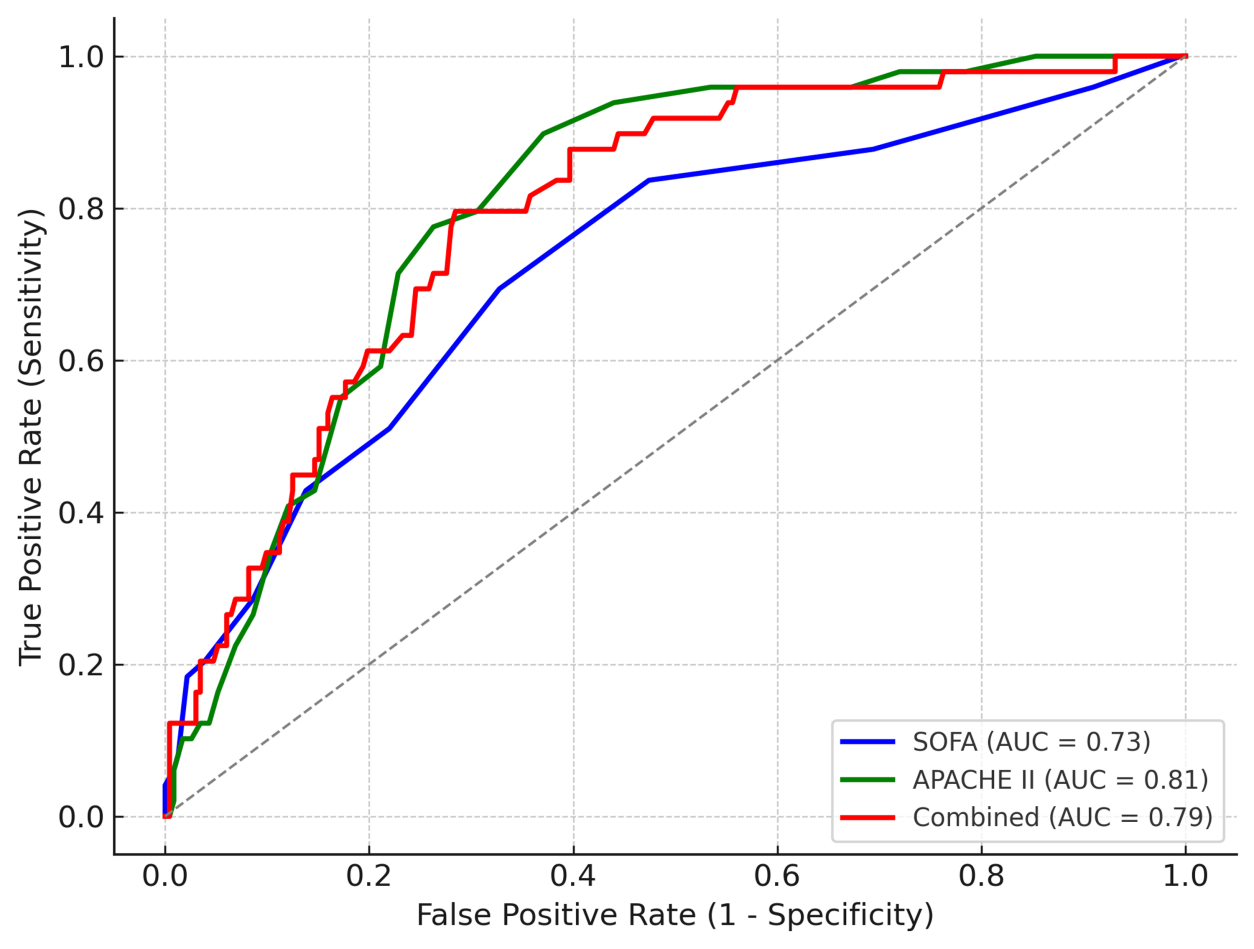
Receiver-operating characteristic (ROC) curves for test cohort models ROC curves for SOFA, APACHE II, and combined (SOFA+APACHE II) logistic regression models in the independent test cohort (n = 281). The curves plot sensitivity against 1–specificity across all possible probability thresholds. Area under the curve (AUC) values are shown in the legend: SOFA = 0.73, APACHE II = 0.81, Combined = 0.79. The diagonal reference line represents chance discrimination (AUC = 0.5).

Three logistic regression models were developed - two based on SOFA and APACHE II scores, while the third model incorporated both scores. In the development cohort, the combined model had the highest discrimination, while in the test cohort, APACHE II performed best. Full AUC values with 95% CIs for all models are presented in Table 4. Receiver operating characteristic curves for the test cohort are shown in Figure 4. All three models consistently lie above the diagonal reference line (AUC=0.5). The AUCs observed are comparable with those reported by Ferreira et al., who found AUCs of 0.74 for SOFA and 0.79 for APACHE II in an intermediate care setting [13].

**Table 4:** Discrimination performance of logistic regression models in development and test cohorts Area under the receiver operating characteristic curve (AUC) with 95% confidence intervals (CI) for SOFA, APACHE II, and combined models. Higher AUC values indicate better model discrimination for predicting 90-day mortality.

Model	Development Cohort AUC (95% CI)	Test Cohort AUC (95% CI)
SOFA	0.78 (0.74 – 0.82)	0.73 (0.64 – 0.81)
APACHE II	0.76 (0.72 – 0.80)	0.81 (0.74 – 0.86)
Combined (SOFA+APACHE II)	0.81 (0.77 – 0.85)	0.79 (0.72 – 0.86)

Calibration

**Figure 5:**
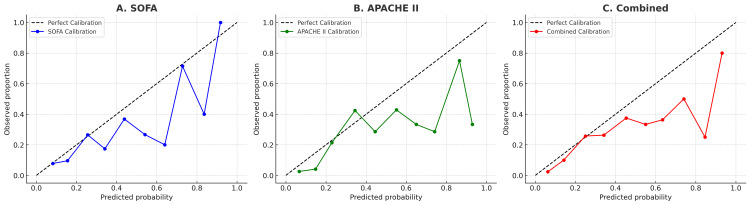
Calibration plots for logistic regression models in the test cohort Calibration of the (A) SOFA, (B) APACHE II, and (C) combined logistic regression models in the independent test cohort (n = 281). The diagonal dashed line represents perfect calibration (predicted risk = observed risk). Circles indicate observed 90-day mortality within deciles of predicted probability, and solid lines show model-predicted probabilities. All three .models tended to overestimate mortality at higher predicted risk levels, with the combined model showing the greatest deviation

Calibration results for the test cohort are summarized in Table 5. All three models had low Brier scores (0.13-0.14), indicating reasonable overall accuracy. The Hosmer-Lemeshow (H-L) test, evaluated with five subgroups, demonstrated evidence of miscalibration. As illustrated in the calibration plots (Figure 5), all models tended to overestimate mortality at higher predicted risk levels. The combined model showed the greatest degree of miscalibration. Given the relatively small test cohort (< 500 patients), the H-L test should be interpreted with caution as it is very sensitive to sample size [14].

**Table 5:** Calibration metrics for logistic regression models in the test cohort Brier scores range from 0 (perfect accuracy) to 1 (poor accuracy), with lower values indicating better calibration. The Hosmer–Lemeshow (H–L) test compares observed versus predicted outcomes across five subgroups; significant p-values indicate miscalibration.

Model	Brier Score	Hosmer–Lemeshow χ²	p-value
SOFA	0.14	12.57	0.006
APACHE II	0.13	19.60	<0.001
Combined (SOFA+APACHE II)	0.14	22.26	<0.001

Probability Threshold Analysis

While two resampling strategies, bootstrap and random 80/20 splits, were analysed in this study, bootstrap resampling was selected as the primary internal validation method. Compared with 80/20 splits, bootstrap uses all observations for both model fitting and out-of-bag evaluation.

The bootstrap-derived median thresholds derived from Youden’s J statistic are reported in Table 6. When the bootstrap-derived thresholds were applied to the independent cohort, they achieved substantially higher sensitivities compared with the conventional 50% cut-off. For example, the combined model identified 88% of deaths at its optimized threshold, compared with only 35% at the 50% cut-off. As expected, the gain in sensitivity was accompanied by reduced specificity, reflecting the trade-off between the two metrics.

**Table 6:** Test cohort performance at default and optimized probability thresholds Performance of SOFA, APACHE II, and combined models in the independent test cohort (n=281). Sensitivity, specificity, positive predictive value (PPV), negative predictive value (NPV), and raw confusion matrix counts (true positives = TP, false positives = FP, true negatives = TN, false negatives = FN) are shown at the conventional 0.50 cut-off and the optimized bootstrap-derived thresholds (10,000 iterations, Youden’s J statistic).

Model	Threshold	Sensitivity	Specificity	PPV	NPV	TP	FP	TN	FN
SOFA	0.50	0.29	0.91	0.42	0.86	14	20	212	35
	0.21	0.69	0.67	0.31	0.91	34	76	156	15
APACHE II	0.50	0.27	0.91	0.39	0.85	13	20	212	36
	0.27	0.78	0.74	0.38	0.94	38	61	171	11
Combined (SOFA + APACHE II)	0.50	0.35	0.89	0.40	0.87	17	26	206	32
	0.19	0.88	0.60	0.32	0.96	43	92	140	6

Negative predictive values remained consistently high across models. Positive predictive values were modest across all models, reflecting the relatively low event rate in the test cohort.

Temporal Stability Analyses

Potential bias introduced by the temporal split between the 2024 development and 2025 test cohorts was evaluated using the rolling-origin temporal validation approach. Sequential 6-month training and 2-month test window advanced monthly across the study period.

Mean test AUCs for SOFA, APACHE II, and the combined model were 0.79, 0.78, and 0.81, respectively (Table 7), with minimal variation across windows. Full rolling-window results are provided in Supplementary Tables 8-10.

**Table 7:** Summary of temporal stability across rolling 6-month training and 2-month testing windows (January 2024 – May 2025)

Model	Mean AUC (Test)	AUC Range	Mean Brier (Test)	Brier Range	Mean Mortality (Test)
SOFA	0.79	0.70 – 0.89	0.13	0.11 – 0.16	19.30%
APACHE II	0.78	0.68 – 0.88	0.13	0.10 – 0.16	19.30%
Combined	0.81	0.73 – 0.90	0.13	0.10 – 0.16	19.30%

## Discussion

This study evaluated the performance of logistic regression models using SOFA, APACHE II, and a combined SOFA-APACHE II framework for predicting 90-day mortality in critically ill adults. Two internal validation approaches-10,000-iteration bootstrap resampling and repeated 80/20 random splits-were used to estimate optimal probability thresholds. Both methods produced remarkably similar threshold values, but bootstrap resampling was selected as the primary approach because it makes full use of the available dataset and provides more stable estimates in modest sample sizes. Model performance was assessed in terms of discrimination and calibration, and findings were evaluated in an independent temporal test cohort.

After deriving optimal probability thresholds using bootstrap resampling in the development cohort, these thresholds were applied to the independent test cohort to assess their clinical impact. Using the conventional 0.50 cut-off, all models showed markedly reduced sensitivity, with a substantial proportion of deaths misclassified as survivors. In contrast, the Youden-optimized thresholds (0.19-0.27 across models) produced much higher sensitivity in the test cohort. For example, the combined model correctly identified nearly 9 out of 10 deaths at its optimized threshold, compared with fewer than 4 out of 10 when using the traditional threshold. Similar results have been reported in prediction modelling literature, where SOFA and APACHE II improved discrimination at lower cut-offs [15].

Across all three models, negative predictive values were consistently high, indicating reliable identification of patients at genuinely low risk. Positive predictive values were modest, which is expected because PPV is mathematically influenced by the frequency of the outcome in the dataset rather than by model performance alone.

In the independent test cohort, APACHE II demonstrated the highest discrimination (AUC 0.81), followed by the combined model (AUC 0.79) and then SOFA (AUC 0.73). These results indicate that, within this cohort, APACHE II was the strongest rank discriminator of mortality risk, while combining SOFA and APACHE II did not improve discrimination relative to APACHE II alone.

These findings are broadly consistent with previous evaluations of longer-term ICU mortality. Oh et al. reported AUCs of 0.732 for SOFA and 0.662 for APACHE II in ventilated patients with multidrug-resistant bacteraemia [16]. Similarly, Minne et al. reported AUC ranges between 0.71 and 0.88 in a systematic review of prognostic models in elderly ICU patients [17]. Although some studies have suggested that combining severity-of-illness indices may offer incremental benefit in specific populations, the present results indicate that such improvements are not universal and may depend on case-mix, cohort characteristics, or the degree of overlap in physiological variables captured by the component scores [18,19].

Calibration assessment provided additional insight into model performance beyond discrimination. Across all three models, predicted and observed mortality aligned closely at lower predicted probabilities, but the calibration plots demonstrated systematic overestimation of risk in the higher-probability range. This pattern likely reflects the limited number of extremely high-risk cases available for model fitting, resulting in greater uncertainty in the upper tail of the predicted distribution. Clinically, high predicted probabilities should therefore be interpreted as prompts for heightened situational awareness - encouraging reassessment of the patient’s physiology, monitoring, and organ support - rather than as literal estimates of mortality risk or automatic triggers for escalation. Routine escalation in response to every high-risk alert is not desirable, as this may contribute to alert fatigue; model outputs should complement, not replace, clinical judgement.

While these findings describe how the models performed in the held-out 2025 cohort, it is equally important to determine whether such performance is consistent across time and case-mix changes. For this reason, a rolling-origin temporal validation was undertaken. Temporal validation demonstrated that all three models maintained stable discrimination and calibration across sequential 6-month training and 2-month testing windows, indicating robustness to temporal shifts in case-mix. Notably, although the combined model showed slightly lower discrimination than APACHE II in the independent test cohort (AUC 0.79 vs 0.81), the rolling-origin analysis revealed a different pattern: the combined model achieved the highest mean test AUC (0.81) and exhibited the greatest temporal stability across windows. This suggests that, while combining the scores did not improve performance in a single held-out cohort, it may offer enhanced robustness for prospective or operational use.

It is important to emphasize that the models developed in this study are not intended for immediate clinical deployment. As with all prediction models, the logistic regression coefficients reflect the characteristics and practice patterns of the population in which they were derived. Major changes in ICU protocols, admission criteria, or treatment pathways could alter these relationships over time, necessitating model updating or recalibration.

This study also has several limitations. The test cohort was modest in size, and the single-centre design limits generalizability. The models are not disease-specific, and performance may vary across clinical subgroups or institutions with different case-mix and resource configurations. Nevertheless, the simplicity and accessibility of SOFA and APACHE II support their potential use as pragmatic risk-stratification tools, particularly in low-resource settings, provided they undergo appropriate external validation.

Future work should include larger multicentre studies to validate these findings across more diverse populations, particularly as calibration and threshold behaviour may vary with case-mix and institutional practice. Further research should also explore disease-specific refinements and expand outcomes beyond mortality alone. Incorporating morbidity-related endpoints-such as ICU length of stay, organ-support trajectories, or complications-would provide a broader assessment of clinical utility.

## Conclusions

The combined SOFA-APACHE II model offers a simple and accessible approach to mortality risk stratification, particularly in low-resource settings where both scores can be calculated at the bedside or implemented using commonly available tools such as spreadsheets and clinical apps. This study shows that reliance on the conventional 0.50 probability threshold markedly reduces sensitivity in unbalanced ICU populations, and that optimizing the probability threshold using the Youden J statistic provides a more clinically meaningful balance of sensitivity and specificity. These findings highlight the value of threshold optimization when applying established severity scores within logistic regression frameworks.

However, even once adequately validated, these models should be used only as adjuncts to clinical judgement rather than as stand-alone prognostic instruments. Before any real-world implementation can be considered, the limitations identified in this study must be addressed, particularly the need for external validation across multiple centres, evaluation in different case-mix and disease-specific subgroups, and confirmation of performance and calibration under varied clinical conditions. Further work in these areas is essential to establish the generalizability and practical readiness of these models for routine clinical use.

## Appendices

Appendix 1. SOFA permission status

The Sequential Organ Failure Assessment (SOFA) score was originally described by Vincent et al. [4] and developed by the European Society of Intensive Care Medicine. In this study, SOFA scores were automatically generated by *Medicus Critical Care* (Mela Solutions Ltd), a licensed electronic medical record (EMR) system used at Walsall Manor Hospital.

The investigator did not manually calculate or reproduce the SOFA scoring table. The score is described in the Methods section with reference to the original source [4]. No additional permission was required for academic use.

Appendix 2. APACHE II Permission Status

The Acute Physiology and Chronic Health Evaluation II (APACHE II) score was originally described by Knaus et al. [1]. In this study, APACHE II scores were automatically generated by *Medicus Critical Care* (Mela Solutions Ltd), a licensed electronic medical record (EMR) system used at Walsall Manor Hospital.

Access to the system is administered by the hospital. Licensing responsibility for APACHE II rests with the vendor. The investigator did not manually calculate or reproduce the APACHE II scoring table. Only the original publication [1] has been cited in the manuscript.

Supplementary Tables

**Table 8:** Rolling 6-month training and 2-month testing temporal validation for the Combined model (January 2024 – May 2025)

Model	Train Start	Train End	Test Start	Test End	Train, n	Test, n	Train Mortality %	Test Mortality %	AUC (Train)	AUC (Test)	Brier (Train)	Brier (Test)
Combined	Jan-24	Jun-24	Jul-24	Aug-24	320	45	24	11	0.80	0.73	0.14	0.12
	Feb-24	Jul-24	Aug-24	Sep-24	335	48	25	15	0.81	0.77	0.14	0.12
	Mar-24	Aug-24	Sep-24	Oct-24	347	51	24	18	0.82	0.81	0.14	0.11
	Apr-24	Sep-24	Oct-24	Nov-24	360	53	21	19	0.81	0.86	0.13	0.11
	May-24	Oct-24	Nov-24	Dec-24	370	50	20	26	0.82	0.90	0.13	0.10
	Jun-24	Nov-24	Dec-24	Jan-25	382	47	20	23	0.83	0.83	0.13	0.11
	Jul-24	Dec-24	Feb-25	Mar-25	395	44	20	14	0.83	0.78	0.12	0.13
	Aug-24	Jan-25	Apr-25	May-25	406	46	21	20	0.82	0.79	0.13	0.11

**Table 9:** Rolling 6-month training and 2-month testing temporal validation for the APACHE II model (January 2024 – May 2025)

Model	Train Start	Train End	Test Start	Test End	Train, n	Test, n	Train Mortality %	Test Mortality %	AUC (Train)	AUC (Test)	Brier (Train)	Brier (Test)
APACHE II	Jan-24	Jun-24	Jul-24	Aug-24	320	45	24	11	0.76	0.75	0.14	0.12
	Feb-24	Jul-24	Aug-24	Sep-24	335	48	25	15	0.77	0.78	0.14	0.12
	Mar-24	Aug-24	Sep-24	Oct-24	347	51	24	18	0.78	0.80	0.14	0.12
	Apr-24	Sep-24	Oct-24	Nov-24	360	53	21	19	0.78	0.84	0.14	0.11
	May-24	Oct-24	Nov-24	Dec-24	370	50	20	26	0.78	0.88	0.13	0.10
	Jun-24	Nov-24	Dec-24	Jan-25	382	47	20	23	0.79	0.83	0.13	0.12
	Jul-24	Dec-24	Feb-25	Mar-25	395	44	20	14	0.80	0.75	0.13	0.14
	Aug-24	Jan-25	Apr-25	May-25	406	46	21	20	0.78	0.78	0.13	0.12

**Table 10:** Rolling 6-month training and 2-month testing temporal validation for the SOFA model (January 2024 – May 2025)

Model	Train Start	Train End	Test Start	Test End	Train, n	Test, n	Train Mortality %	Test Mortality %	AUC (Train)	AUC (Test)	Brier (Train)	Brier (Test)
SOFA	Jan-24	Jun-24	Jul-24	Aug-24	320	45	24	11	0.78	0.70	0.15	0.13
	Feb-24	Jul-24	Aug-24	Sep-24	335	48	25	15	0.80	0.75	0.15	0.13
	Mar-24	Aug-24	Sep-24	Oct-24	347	51	24	18	0.80	0.77	0.15	0.13
	Apr-24	Sep-24	Oct-24	Nov-24	360	53	21	19	0.79	0.82	0.14	0.12
	May-24	Oct-24	Nov-24	Dec-24	370	50	20	26	0.79	0.89	0.14	0.11
	Jun-24	Nov-24	Dec-24	Jan-25	382	47	20	23	0.81	0.86	0.13	0.12
	Jul-24	Dec-24	Feb-25	Mar-25	395	44	20	14	0.83	0.77	0.13	0.14
	Aug-24	Jan-25	Apr-25	May-25	406	46	21	20	0.81	0.79	0.14	0.12
